# Sub-Acute Toxicity Study of Graphene Oxide in the Sprague-Dawley Rat

**DOI:** 10.3390/ijerph13111149

**Published:** 2016-11-17

**Authors:** Yingbo Li, Yan Wang, Liu Tu, Di Chen, Zhi Luo, Dengyuan Liu, Zhuang Miao, Gang Feng, Li Qing, Shali Wang

**Affiliations:** 1 Cerebrovascular Diseases Laboratory, Institute of Neuroscience, Chongqing Medical University, Chongqing 400016, China; xiaosui19781127@163.com (Y.L.); 15223071977@163.com (Y.W.); xiangping.hh@gmail.com (L.T.); chendidi@hotmail.com (D.C.); 2 Preventive Medicine, School of Public Health and Management, Chongqing Medical University, Chongqing 400016, China; LZ292874810@Hotmail.com (Z.L.); 18375750155@163.com (D.L.); 15396919928@163.com (Z.M.); 3 Pediatric Specialty of Clinical Medicine, Academy of Pediatrics, Chongqing Medical University, Chongqing 400016, China; 18324181946@163.com; 4 The First Clinical College of Chongqing Medical University, Chongqing 400016, China; 18883938786@163.com

**Keywords:** graphene oxide, carbon nano-material, sub-acute toxicology

## Abstract

Graphene oxide (GO) is an oxidized derivative of graphene used in biotechnology and medicine. The safety of GO is uncertain, so we evaluated its toxicity in male rats. Rat tail veins were injected with 2.5, 5, or 10 mg/kg GO for seven days and behavioral patterns, pathology, and tissue morphology were assessed. Data show that behaviors were not altered according to an open field test and a functional observational battery test, but histopathological analysis indicated that GO caused inflammation of the lung, liver, and spleen. GO also reduced cholesterol, high density lipoprotein (HDL), and low density lipoprotein (LDL). No other organs were modified. Thus, high concentrations of GO are toxic for the lung, liver, and spleen, but the mechanism by which this occurs requires more study.

## 1. Introduction

Carbon nanomaterials exist in nature in multiple forms and are used in science and technology as materials for drug/gene delivery [1], cell imaging [2], biosensing [3], and cancer therapy [4]. Graphene was first isolated from graphite in 2004 [5] and graphene oxide (GO) is an oxidized derivative of graphene ranging from 10 nm to 1 μm with numerous hydroxyl, carbonyl, and carboxyl epoxy groups randomly distributed on the surface [6,7], giving it unique properties. Recently, due to its physicochemical properties, GO has been be used for immobilization of various biomolecules, and it has potential uses in drug delivery [8,9]. GO has high surface area and a strong adsorption capacity for drugs and maintains drug stability without altering biological activity, far more than other nanomaterials [10,11,12]. Studies show that GO with lateral sizes from 200 μm to 500 nm can serve as a means of drug delivery [13,14].

Although the biological applications of GO have not been well-studied, it has extraordinary biological action. Under ultrasonic treatment, nanoscale GO (lateral size 1–100 nm) will form a stable graphite oxide colloid and can be suspended in water and used to measure DNA and proteins [15,16]. GO (lateral size around 10 μm) has antibacterial activity [17] and GO which is around 5–30 μm in lateral size could promote the differentiation of embryonic stem cells [18]. This shows that GO which have different sizes may have different biological characteristics. With chemical modification, more functions can be ascribed to GO. Carboxylated GO disperses well in water [19]. Methacrylate GO (MeGO) which disperses in methacrylated gelatin (GelMA) solution can be used to measure encapsulated fibroblast viability and proliferation [20]. PEGylated (polyethylene glycol functionalized) GO can target tumors and be used for photothermal therapy [21].

Studies show that nanomaterials can disperse within the body (except for the brain) and that it is toxic, able to induce cell death or alter gene expression [22,23,24]. Currently, carbon nanomaterial biosecurity is under consideration due to its widespread use in biology. Studies have focused on graphene toxicity in vivo and in vitro. Some reports suggest that GO is safe [25,26] and others suggest otherwise. After intravenous administration, GO (lateral size 10–800 nm, 10 mg/kg) may lead to granuloma formation [25] and others reported that GO (lateral size 100–500 nm, 20 mg/kg in vivo, 100 μg/mL in vitro) can induce mutagenesis in vitro and in vivo [27]. These studies may imply that GO with a lateral size of around 10–800 nm will lead to tissue injury. However, GO (lateral size 100–800 nm, 200 μg/mL) is generally non-toxic at the cellular level in A549 cells [28]. Wang’s group reported that GO sheet (at a thickness of 1 nm) administration in mice induced chronic toxicity and lung granulomas [22]. Short-term repeated GO (which thickness around 1 nm) exposure can cause reversible eye damage via oxidative stress [29]. All these studies show that GO with different sizes and concentration scan lead to different effects on the different organs or tissues. At present, GO has not been assessed in all major organs. In our study, we try to study the effect of GO with a lateral size that is between 100–500 nm on rats; we analyzed behavior, histopathology of animal organs, and serum biochemistry to elucidate the toxicity of GO in vivo, in an effort to illuminate the toxicology of GO with lateral sizes between 100–500 nm.

## 2. Materials and Methods

### 2.1. GO Characterization

GO dispersion (2 mg/mL, Lot number: FG0016) was purchased from First Graphene (First Graphene, Suzhou, China). GO samples were characterized with a transmission electron microscope (TEM) (Toshiba 7005, Tokyo, Japan), Fourier transform infrared spectroscopy (FTIR, Avatar 370, Thermo Nicolet, San Diego, CA, USA), and ultraviolet scanning (UV, UV7504, CANY, Shantou, Guangdong, China). For TEM detection, the GO was placed on a copper grid, and subjected to natural drying. To get the average size of the GO, three GO samples were made and randomly collected five images for each sample. GOs were dispersed in ultra-pure water to prepare the stock solution (1.0 mg/mL). Before application, the solution was sonicated for 1 h (75 kHz, 100 W), disinfected by pasteurization at 70 °C for 30 min, and then stored at 4 °C.

### 2.2. Experimental Design

Adult male Sprague-Dawley rats (eight to nine weeks-of-age, 100 ± 5 g) were purchased from the Animal Department of Chongqing Medical University (Chongqing, China) and randomized to four treatment groups (*n* = 12/group). Animals were allowed to acclimate for seven days prior to treatment and then exposed to GO (2.5, 5, and 10 mg/kg/day) via tail vein injection at 9 AM daily. Control animals were given the same volume of saline. Efforts were made to minimize the number of animals used and the suffering of the animals selected. All experimental procedures were performed under the guidelines of the Experimental Laboratory Animal Committee of Chongqing Medical University and were in strict accordance with the principles and guidelines of the National Institutes of Health Guide for the Care and Use of Laboratory Animals (NIH Publications No. 80-23). The animal protocols used in this work were evaluated and approved by the Experimental Laboratory Animal Committee of Chongqing Medical University (Protocol 2015-051).

### 2.3. Body Weight and Food Consumption

Rats were housed in polycarbonate cages (*n* = 4/cage) and maintained in a controlled atmosphere with a cyclic 12 h dark/12 h light cycle, a temperature of 22 ± 2 °C and 50%–70% humidity. Pelleted and nutritionally balanced food and water were available ad lib. After three days of adaptive feeding, animal weight and feed consumption were measured and recorded at 9 AM daily.

### 2.4. Open Field Test

Open Field Test (OFT) was used to assess locomotor activity and monitored with a camera on days 0 and 7. Animal activity was measured in an 80 cm × 80 cm × 40 cm black-painted cube without a ceiling. Chambers of the cube were divided into 25 squares using white paint. The center (30 cm × 30 cm) was outlined with electrical tape (Tartan 1710, ShengHua, Chengdu, China). The camera was mounted over the middle square (Panasonic, Tokyo, Japan). Scores were calculated as the duration spent rearing (standing upright on hind legs), the number of crossings across grid lines (with at least three paws), number of crossings of the inner square, and grooming. 

### 2.5. Functional Observational Battery (FOB) Test

FOB observations were recorded on days 0 and 7. Testing was performed by the same technicians, whenever possible, who were blinded to treatment groups. The FOB was performed in a sound-protected room equipped with a white noise generator (70 ± 10 dB) and home cage observations were performed in the common animal room. All animals were observed as indicated in Table 1 based on previously developed protocols [30,31].

### 2.6. Organ Coefficient

Gross pathology was conducted by visual inspection during necropsy. Brain, heart, lungs, kidneys, spleen, liver, thymus gland, adrenal gland, prostate gland, testis, epididymis were excised and weighed. Relative weight of each organ was calculated based on the final body weight measured on that day. An organ coefficient was calculated (organ coefficient (g/100 g) = organ weight/rat body weight × 100).

### 2.7. Clinical Pathology

Blood samples for hematology and serum chemistry were collected from animal hearts at necropsy (day 8) after fasting. Erythrocytes, hematocrit, mean red cell volume, hemoglobin, mean corpuscular hemoglobin, leucocytes, lymphocytes, platelets, prothrombin time, activated partial thromboplastin time, and thrombin time were measured on an Abbott CELL-DYN 3500 (Mairui, Shenzhen, China) according to the manufacturer’s manual. Serum albumin/globulin, alanine transaminase, aspartate transaminase, alkaline phosphatase, albumin, lactate dehydrogenase, total protein, glucose, glutamyltransferase, triglycerides, cholesterol, high density lipoprotein (HDL), low density lipoprotein (LDL), urea, and creatinine were measured with a Hitachi Model 912 (Hitachi, Tokyo, Japan) using application codes provided by the manufacturer and reagents provided by Boehringer Mannheim (Indianapolis, IN, USA).

### 2.8. Histopathological Evaluation

Four rats of each group were anesthetized and perfused with saline followed by 4% paraformaldehyde solution. Then organs were harvested and immersed in 4% paraformaldehyde. Organs were embedded in paraffin and cut into 4 μm sections. Sections were mounted to glass slides and paraffin-embedded sections were dewaxed with xylene, rehydrated through an ethanol series (absolute, 95%, 90%, 80%, and 70%) and washed with water. Hematoxylin and eosin (H & E) staining was performed on paraffin sections of brain, heart, liver, lung, spleen, kidney, and lymph gland.

### 2.9. Lactate Dehydrogenase (LDH) in Bronchoalveolar Lavage Fluid (BALF)

BALF was obtained using 50 mL 0.9% NaCl which was instilled via the trachea into the lungs and immediately withdrawn. BALF was centrifuged at 1000× *g* for 5 min at 4 °C, and cell-free supernatant was collected for LDH measurement with an assay kit (Nanjing Institute of Jiancheng Biological Engineering, Nanjing, China) according to the manufacturer’s instructions [32].

### 2.10. Statistical Analysis

SPSS 17.0 software was used for all statistical calculations. All data are presented as means with standard deviations (means ± SD). The significant differences between groups were compared using One-way analysis of variance (ANOVA). Statistically significant mean differences were evaluated using ANOVA followed by the Student-Newman-Keuls test for multiple comparisons. ANOVA with *p*-values less than 0.05 were considered as statistically significant. 

## 3. Results

### 3.1. Characterization of GO

**Figure 1:**
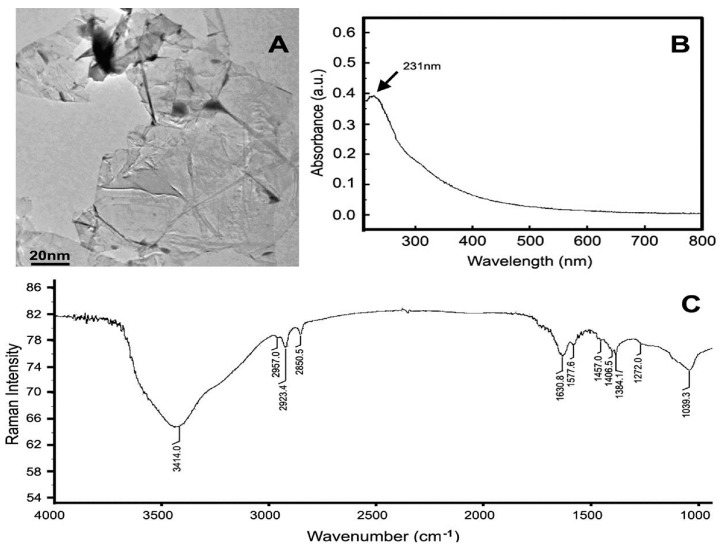
Characterization of graphene oxide (GO). (**A**) Transmission electron microscope (TEM) shows GOs 100–500 nm; (**B**) ultraviolet (UV) scanning shows GO absorption peak at 231 nm; (**C**) Fourier transform infrared spectroscopy FTIR analysis suggests obvious characteristic peaks: 3414 cm^−1^, 1630 cm^−1^, 1040 cm^−1^, and 1577 cm^−1^.

Representative TEM images of the GO samples are shown in Figure 1A. Most GO sheets exist as irregular single layers or a few layers of thickness. The TEM shows the lateral sizes of GOs were 100–500 nm. UV scanning data show a GO absorption peak at 231 nm (Figure 1B). Raman analysis suggests the existence of hydroxyl (3414 cm^−1^) and carboxyl epoxy groups (1630 cm^−1^), and a C=C bond (1040 cm^−1^ and 1577 cm^−1^) in GO samples.

### 3.2. General Observation, Body Weight, and Food Consumption

**Figure 2:**
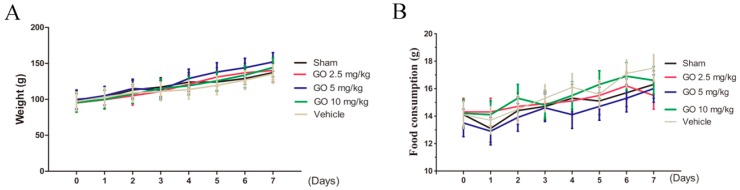
Body weight and food consumption. (**A**) Food consumption (g/animal/day) was observed for all GO doses. No differences were noted; (**B**) Rat weight was measured daily and these did not differ among groups. *n* = 5 rats, Mean ± SD.

No sign of morbidity or mortality was observed during the study. Daily examination of rat physical condition and wellbeing indicated no toxicity throughout the study period. Animal weight and food consumption was monitored over seven days and recorded. No significant differences in food consumption or animal weight among any group were noted (*p* > 0.05 for both). See Figure 2 for data.

### 3.3. Neurobehavioral Assessment

Crossings of inner squares, rearing, and grooming, were measured in the open field test and no difference was noted in any group during the study (Table 2, *p* > 0.05). GO did not affect locomotor activity and exploratory behavior and effects were not observed for movement, muscle tone, response, excitability, and independent activity (Table 3, *p* > 0.05).

### 3.4. Morphology, Weight, and Organ Assessment

The morphology of brain, heart, lung, kidney, thymus, adrenal gland, prostate, testis, and epididymis in all dose groups were not abnormal compared to controls, and organs were smooth and uniform. Organ weights did not differ among groups as well (Table 4). The lungs of GO 10 mg/kg treated animals were slightly greater than controls but this did not reach statistical significance.

### 3.5. Pathological Changes

**Figure 3:**
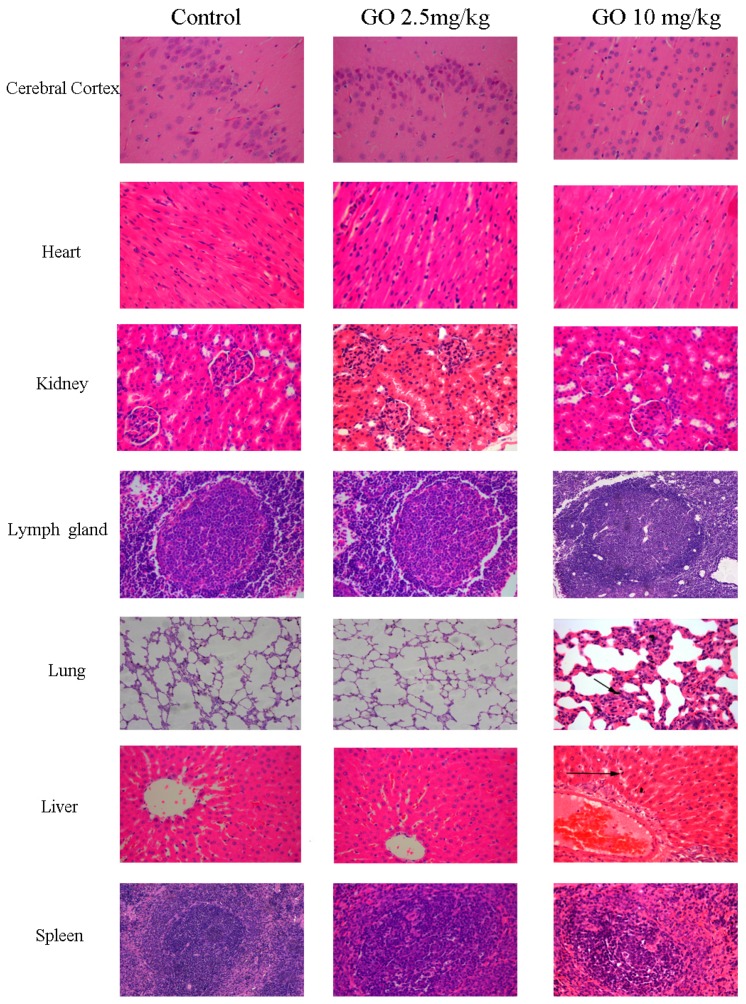
GO can induce an inflammatory response in lungs, liver, spleen. Rats treated with (2.5, 5, 10 mg/kg) did not undergo changes to the cerebral cortex, heart, kidney, and lymph glands, and there was no GO deposition in these organs. Only the highest dose of GO injured the liver, lungs, and spleen which were hyperemic and inflamed after seven days. Some GO aggregated in the lungs and liver. Black arrows indicate GO deposition.

H&E was used to visualize pathological changes of organs (Figure 3). There were no obvious structural changes in organs with respect to GO deposition in the cerebral cortex, heart, kidney, and lymph glands. Lungs, liver, and spleens were different in animals treated with GO 10 mg/kg. Pulmonary capillaries were hyperemic, alveolar walls were thicker, and less alveolar space was observed along with granulomatous reactions. GO appeared in the lung as shown in Figure 3. The liver capillaries were hyperemic, and the hepatic sinusoid and central veins were enlarged. GO particles were scattered in the hepatic sinusoid and the spleen capillaries were hyperemic with activated lymphocytes. GO particles were seen in the spleen.

### 3.6. Hematology, Serum Chemistry, and BALF

There were no GO-related effects on hematology (Table 5). GO treated animals did not differ from controls. Serum chemistry data showed that only LDH differed between treated and control animals, but this was not statistically significant. Thus, liver or renal function was not impaired. Cholesterol, HDL, and LDL were lower in the highest GO treated group (Table 6). LDH in Bronchoalveolar Lavage Fluid (BALF) was greater in the 10 mg/kg GO group but not in other groups, compared to controls. Thus, GO was toxic to the lungs at the highest concentration used [33].

## 4. Discussion

In our study, Sprague-Dawley rats were exposed to GO via tail vein injection and then we assessed behavior and histopathology of organs along with serum biochemistry to estimate the injury or change to organs. Table 2 and Table 3 show that functional and locomotor behaviors were unchanged, likely because GO cannot cross the blood brain barrier. Indeed, no brain changes were noted with H & E staining and GO deposition was not observed. These observations differ from studies of gold nanoparticles which can pass through the blood brain barrier [34]. Thus, GO may not be toxic to the central nervous system. We also noted no injury to the heart or kidneys (Figure 3), and these data are supported by serum chemistry data in Table 6. Creatine kinase and cardiac troponin, cardiac injury markers [35], were measured and these did not differ between treated animals and controls. Creatine was also not elevated. Thus, GO may not be toxic to the brain, heart, and kidney.

**Figure 4:**
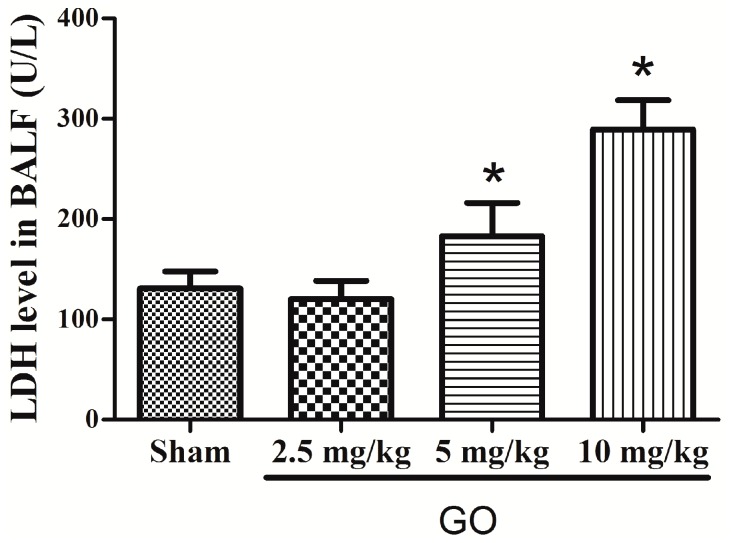
GO induces LDH in bronchoalveolar lavage fluid (BALF). LDH was greater after GO (10 mg/kg) treatment compared to controls but lower doses of GO were not different than controls. * *p* < 0.05 compared with groups, *n* = 5.

Figure 3 shows that GO (10 mg/kg) for seven days caused inflammation in the lungs, liver, and spleen, but this did not occur for the other two treatments (2.5 and 5 mg/kg). Lactate dehydrogenase (LDH) in bronchoalveolar lavage fluid (BALF) was assessed, and Figure 4 shows that at the highest GO treatment, LDH was greater than in controls, indicating that GO can injure the lung and cause apoptosis. Serum LDH was not different from controls and other serum markers were not abnormal. Thus, high-dose GO can injure the lung. GO has been reported to be able to enter rat circulation and organs via the caudal vein, reaching the lungs and later being taken up by mononuclear phagocytes in the liver and spleen [36]. Liu reported that GO (10–500 nm, 10 mg/kg) distributed to the lungs and liver [37], which agrees with our data. We found that some GO aggregated in the lungs and liver after GO (10 mg/kg) injection (Figure 3). GO’s effects are dose related and others have reported that particle size may also contribute to distribution and toxicity [22]. GO is not easily degraded in the body, so it may accumulate and be identified by immune cells thereby activating the reticuloendothelial system [12] which stimulates fibroblast proliferation and non-inflammatory granulomas [38].

We also noted that serum cholesterol, HDL, and LDL were reduced after the highest exposure to GO, indicating alterations in lipid metabolism. Whether GO can bind to these lipids and absorb them for excretion is uncertain and requires more study. GO with size around 100 nm has a surface-active group [16]; as such, it may be able to combine with serum lipids, and this may require further study.

## 5. Conclusions

Male rats exposed to GO for seven days experienced inflammation of the lung, liver, and spleen, with effects being concentration dependent. GO is apparently not toxic for other organs. This represents preliminary information about GO toxicity in rats and more verification and mechanistic elucidation are required before GO is safely used for biomedical applications.

## Figures and Tables

**Table 1:** Neurobehavioral assessment.

Observations	Parameters
Open Field Test (OFT)	Numbers of crossing Number of rearing Inner squares crossed Number of grooming
Functional Observational Battery (FOB)	Body temperature, Urination Defecation, Grooming Backing, Hind limb foot splay Muscle tone, Convulsions/tremors Clonic movement, Catalepsy Gait score, Activity score Arousal, Bizarre/stereotypic Red/crusty deposits, Approach response Touch response, Startle response Tail pinch response

**Table 2:** Locomotor activity of rats in the open field before or after GO injection.

Locomotor Activity	Sham		GO 2.5 mg/kg		GO 5 mg/kg		GO 10 mg/kg	
	0 Day	7 Day	0 Day	7 Day	0 Day	7 Day	0 Day	7 Day
Numbers of crossings	90.0 ± 8.0	83.25 ± 6.9	86.75 ± 6.8	80.5 ± 7.8	84.5 ± 7.8	74.5 ± 9.2	79.25 ± 9.8	77.75 ± 9.71
Number of rearings	16.5 ± 4.0	17.5 ± 1.3	17.0 ± 5.8	16.2 ± 3.9	19.0 ± 4.4	15.5 ± 0.9	17.75 ± 4.3	14.5 ± 1.29
Inner squares crossed	26.5 ± 11.9	26.35 ± 6.9	24.71 ± 5.2	29.3 ± 8.7	23.5 ± 6.1	28.0 ± 7.8	28.0 ± 9.9	23.1 ± 7.27
Number of groomings	2.3 ± 0.9	2.5 ± 1.3	2.7 ± 1.0	2.7 ± 1.2	2.5 ± 0.9	3.2 ± 1.2	3.2 ± 0.9	2.5 ± 1.1

*p* > 0.05 compared with sham groups, *n* = 8 rats, Mean ± SD.

**Table 3:** Functional Observational Battery (FOB) of rats before or after GO injection.

Functional Observational	Score	Sham		GO 2.5 mg/kg		GO 5 mg/kg		GO 10 mg/kg	
		0 Day	7 Day	0 Day	7 Day	0 Day	7 Day	0 Day	7 Day
Body temperature (°C)	-	36.6 ± 0.23	36.48 ± 0.13	36.78 ± 0.38	36.53 ± 0.17	36.6 ± 0.36	36.43 ± 0.13	36.8 ± 0.58	36.4 ± 0.18
Urination	-	2.45 ± 0.96	3.05 ± 0.96	2.0 ± 0.82	3.0 ± 0.82	2.75 ± 0.96	3.0 ± 0.82	2.0 ± 0.82	2.75 ± 1.26
Defecation	-	3.50 ± 1.0	5.0 ± 0.82	3.50 ± 1.29	4.0 ± 0.82	3.5 ± 1.29	4.5 ± 1.29	3.25 ± 1.25	3.75 ± 0.96
Grooming	-	1.0 ± 0.82	1.0 ± 0.82	1.75 ± 0.96	1.0 ± 0.82	1.75 ± 0.96	1.5 ± 0.58	1.25 ± 0.5	1.0 ± 0.82
Backing	-	9.37 ± 3.18	8.85 ± 4.10	8.74 ± 3.47	7.59 ± 5.34	10.00 ± 6.42	9.97 ± 3.55	9.67 ± 4.34	7.07 ± 3.72
Hind limb foot splay	-	7.77 ± 0.93	8.1 ± 0.13	7.78 ± 0.52	7.77 ± 0.73	8.17 ± 0.59	8.14 ± 0.15	8.23 ± 0.13	7.9 ± 0.31
Muscle tone	0	8/8	8/8	8/8	8/8	8/8	8/8	8/8	8/8
	1								
	2								
Convulsions/tremors	0	8/8	8/8	8/8	8/8	8/8	8/8	8/8	8/8
	1								
	2								
Clonic movement	0	8/8	8/8	8/8	8/8	8/8	8/8	8/8	8/8
	1								
	2								
Catalepsy	0	8/8	8/8	8/8	8/8	8/8	8/8	8/8	8/8
	1								
	2								
Gait score	0								
	1	1/8		1/8			2/8		
	2	7/8	8/8	7/8	8/8	8/8	6/8	8/8	8/8
Activity score	0								
	1	6/8	6/8	8/8	7/8	7/8	8/8	6/8	8/8
	2	2/8	2/8		1/8	1/8		2/8	
Arousal	0			1/8					
	1	8/8	8/8	7/8	8/8	7/8	8/8	8/8	8/8
	2					1/8			
Bizarre/stereotypic	0	8/8	8/8	8/8	8/8	8/8	8/8	8/8	8/8
	1								
	2								
Red/crusty deposits	0	8/8	7/8	8/8	8/8	8/8	8/8	8/8	8/8
	1		1/8						
	2								
Approach response	0								
	1			2/8	1/8		1/8	1/8	
	2	8/8	8/8	6/8	7/8	8/8	7/8	7/8	8/8
Touch response	0								
	1	1/8		2/8	1/8		1/8		
	2	7/8	8/8	6/8	7/8	8/8	7/8	8/8	8/8
Startle response	0								
	1		2/8			1/8			
	2	8/8	6/8	8/8	8/8	7/8	8/8	8/8	8/8
Tail pinch response	0								
	1	8/8	8/8	8/8	8/8	8/8	8/8	8/8	8/8
	2								

*p* > 0.05 compared with sham groups, *n* = 8 rats, Mean ± SD.

**Table 4:** Organ weights of rats following injection of GO in the seven-day subchronic toxicity study.

Organ Weights	Sham	GO 2.5 mg/kg	GO 5 mg/kg	GO 10 mg/kg
Brain				
Absolute organ weight (g)	1.68 ± 0.04	1.72 ± 0.05	1.75 ± 0.06	1.68 ± 0.06
organ coefficient %	1.30 ± 0.12	1.32 ± 0.12	1.34 ± 0.12	1.29 ± 0.16
Heart				
Absolute organ weight (g)	0.55 ± 0.05	0.48 ± 0.05	0.52 ± 0.04	0.5 ± 0.07
organ coefficient %	0.43 ± 0.05	0.36 ± 0.05	0.37 ± 0.03	0.38 ± 0.03
Liver				
Absolute organ weight (g)	4.36 ± 0.14	4.37 ± 0.18	4.04 ± 0.37	3.83 ± 0.42
organ coefficient %	3.33 ± 0.36	3.34 ± 0.39	3.03 ± 0.3	2.99 ± 0.09
Spleen				
Absolute organ weight (g)	0.38 ± 0.06	0.39 ± 0.05	0.44 ± 0.03	0.4 ± 0.04
organ coefficient %	0.31 ± 0.06	0.28 ± 0.03	0.30 ± 0.03	0.31 ± 0.06
Lung				
Absolute organ weight (g)	0.84 ± 0.12	0.79 ± 0.04	0.82 ± 0.16	0.95 ± 0.18
organ coefficient %	0.67 ± 0.08	0.58 ± 0.07	0.67 ± 0.06	0.71 ± 0.06
Kidney				
Absolute organ weight (g)	0.03 ± 0.009	0.04 ± 0.005	0.03 ± 0.005	0.031 ± 0.005
organ coefficient %	0.86 ± 0.08	0.85 ± 0.09	0.82 ± 0.04	0.80 ± 0.03
Thymus gland				
Absolute organ weight (g)	0.26 ± 0.07	0.33 ± 0.043	0.32 ± 0.014	0.33 ± 0.038
organ coefficient %	0.22 ± 0.05	0.27 ± 0.05	0.25 ± 0.02	0.25 ± 0.03
Adrenal gland (×100)				
Absolute organ weight (g)	3.23 ± 0.9	4.22 ± 0.52	3.08 ± 0.48	3.1 ± 0.47
organ coefficient %	2.08 ± 0.45	2.49 ± 0.44	2.17 ± 0.41	2.17 ± 0.25
Prostate gland				
Absolute organ weight (g)	0.87 ± 0.04	0.81 ± 0.04	0.92 ± 0.16	0.95 ± 0.15
organ coefficient %	0.08 ± 0.04	0.07 ± 0.02	0.07 ± 0.02	0.05 ± 0.02
Testis				
Absolute organ weight (g)	1.05 ± 0.26	1.19 ± 0.18	1.14 ± 0.19	1.11 ± 0.06
organ coefficient %	0.86 ± 0.23	0.85 ± 0.09	0.88 ± 0.17	0.94 ± 0.14
Epididymis				
Absolute organ weight (g)	0.7 ± 0.077	0.77 ± 0.19	0.82 ± 0.17	0.79 ± 0.06
organ coefficient %	0.57 ± 0.11	0.65 ± 0.15	0.64 ± 0.08	0.61 ± 0.03

**Table 5:** Routine blood indexes of rats following injection of GO in the seven-day subchronic toxicity study.

Parameter	Sham	GO 2.5 mg/kg	GO 5 mg/kg	GO 10 mg/kg
RBC (×10^12^/L)	4.5 ± 0.1	4.7 ± 0.17	4.67 ± 0.23	4.36 ± 0.24
HCT (%)	33.3 ± 1.25	34.9 ± 1.6	33.68 ± 1.81	31.9 ± 1.67
MCV (fL)	75.75 ± 3.3	74.5 ± 2.08	72.25 ± 1.26	73.25 ± 1.89
Hb (g/L)	101.25 ± 2.99	105.75 ± 4.92	103.5 ± 4.65	97.5 ± 6.35
MCH (pg)	22.75 ± 0.96	22.5 ± 0.58	22.25 ± 0.5	22.5 ± 0.58
MCHC (g/L)	304 ± 3.56	303.25 ± 2.06	307 ± 4.08	306 ± 4.24
WBC (×10^9^/L)	4.88 ± 0.79	6.75 ± 1.63	5.23 ± 0.59	5.68 ± 1.48
LYM (×10^9^/L)	3.7 ± 0.76	5.5 ± 1.44	4 ± 0.88	4.03 ± 0.72
LYM (%)	75.6 ± 5.5	81.55 ± 4.9	75.38 ± 9.8	71.95 ± 6.65
PLT (×10^9^/L)	1034.5 ± 57.36	1075.5 ± 78.8	1053.25 ± 76.5	964.25 ± 131.02
PLT (%)	0.51 ± 0.03	0.52 ± 0.04	0.5 ± 0.04	0.46 ± 0.07
PT (s)	15.28 ± 0.61	15.08 ± 0.53	15 ± 0.63	15.58 ± 0.71
APPT (s)	27.4 ± 3.69	24.6 ± 1.53	25.05 ± 1.63	23.6 ± 8.41
TT (s)	31.93 ± 5.81	27.5 ± 5.94	33.38 ± 7.63	22.65 ± 13.32

*p* > 0.05 compared with sham group, *n* = 8 rats, Mean ± SD; RBC: Red blood cells; HCT: Hematocrit; MCV: Mean red cell volume; Hb: Hemoglobin; MCH: Mean corpuscular hemoglobin; MCHC: Mean corpuscular hemoglobin concentration; WBC: White blood cells; LYM: Lymphocyte; PLT: Platelets; PT: Prothrombin time; APPT: Activated partial thromboplastin time; TT: Thrombin time.

**Table 6:** Clinical chemistry parameters in rats following injection of GO in the seven-day subchronic toxicity study.

Parameter	Sham	GO 2.5 mg/kg	GO 5 mg/kg	GO 10 mg/kg
ALT (U/L)	61.95 ± 9.93	58.35 ± 9.26	58.58 ± 14.34	55.98 ± 6.16
AST (U/L)	140.7 ± 35.24	125.2 ± 16.74	121.5 ± 14.57	119.7 ± 16.07
ALP (U/L)	440.7 ± 74.79	437.4 ± 81.59	385.5 ± 30.51	362.7 ± 52.21
ALB (g/L)	34.7 ± 0.88	34.38 ± 2.97	36.03 ± 2.53	39.4 ± 1.19
LDH (U/L)	269.25 ± 14.66	289.25 ± 10.5	271 ± 7.53	309.5 ± 21.95
TP (g/L)	53.7 ± 1.13	50.95 ± 2.62	52.6 ± 2.3	53.5 ± 3.0
A/G	1.83 ± 0.06	2.09 ± 0.25	1.86 ± 0.31	1.83 ± 0.26
GLU	7.78 ± 0.86	8.65 ± 0.87	7.2 ± 1.13	8.78 ± 2.19
GGT (U/L)	85.9 ± 12.89	79.3 ± 5.79	78.93 ± 2.35	94.6 ± 16.9
TG (mmol/L)	0.7 ± 0.38	0.55 ± 0.21	0.35 ± 0.13	0.28 ± 0.1
CHO (mmol/L)	2.18 ± 0.1	2.05 ± 0.19	2.15 ± 0.24	1.55 ± 0.1 *
HDL (mmol/L)	1.05 ± 0.08	1.11 ± 0.08	1.09 ± 0.08	0.74 ± 0.06 *
LDL (mmol/L)	1.03 ± 0.06	0.93 ± 0.07	1 ± 0.12	0.71 ± 0.06 *
Urea (mmol/L)	3.73 ± 1.15	3.4 ± 1.88	2.83 ± 0.52	3.3 ± 0.99
Cr (µmol/L)	31 ± 4.08	31.5 ± 6.86	35.25 ± 5.12	32.75 ± 5.06
CK (U/L)	93 ± 25.14	107 ± 19.33	91 ± 21.32	115 ± 17.31
cTn (μg/L)	0.09 ± 0.01	0.08 ± 0.01	0.09 ± 0.01	0.11 ± 0.01

* *p* < 0.05, compared with the sham group, *n* = 4 rats, Mean ± SD. A/G: Albumin/Globulin; ALT: Alanine transaminase; AST: Aspartate transaminase; ALP: Alkaline phosphatase; ALB: Albumin; LDH: Lactate dehydrogenase; TP: Total protein; GLU: Glucose; GGT: Glutamyltransferase; TG: Triglycerides; CHO: Cholesterol; HDL: High density lipoprotein; LDL: Low density lipoprotein; Cr: Creatinine; CK: Creatine Kinase; cTn: cardiac troponin.
